# Enteral feed absorption during therapeutic hypothermia following out-of-hospital cardiac arrest

**DOI:** 10.1186/cc9797

**Published:** 2011-03-11

**Authors:** C Smith, J Nolan, M Williams

**Affiliations:** 1 Royal United Hospital, Bath, UK

## Introduction

Enteral feeding is the preferred nutrition method in critically ill patients, with early administration leading to improved outcome [1]. There are no studies documenting the feasibility of enteral feeding during therapeutic hypothermia following cardiac arrest and, in our experience, many intensive care clinicians withhold enteral feed during the hypothermic period.

## Methods

Data were collected retrospectively from patients admitted to the Royal United Hospital ICU for therapeutic hypothermia following out-of-hospital cardiac arrest between 2002 and 2008. We recorded the total enteral feed input, total volume of gastric aspirate, total volume of gastric aspirate that was discarded and the number of vomiting episodes for 72 hours. The first 24 hours was the period of cooling, the second 24 hours included 14 hours of re-warming and 10 hours of normothermia, and the third 24 hours was normothermia. Feed balance was calculated by subtracting the volume of discarded aspirate from the volume of enteral input.

## Results

Thirty-two patients were included in the study. The median feed balance, percentage of patients with a positive feed balance, number of vomiting episodes and percentage of patients vomiting for each day is given in Table 1.

**Table 1:** Median feed balance (MFB), positive feed balance (PFB) and vomiting episodes

Day	MFB (ml) (IQR)	PFB (*n *(%))	Vomiting (*n *(%))
1	265 (53 to 788)	25 (78.1)	8 (9.4)
2	400 (69 to 1,229)	24 (82.6)	6 (10.3)
3	572 (122 to 1,131)	22 (84.6)	6 (7.7)

## Conclusions

Absorption of enteral feed increased with increasing core temperature. Even during hypothermia, the median feed balance was positive by 265 ml and 78% of patients had a positive feed balance and 9.4% of patients experienced vomiting. This implies that at a core temperature of 33°C there is sufficient gastrointestinal function to enable some enteral feed to be absorbed in most patients without a significant increase in vomiting. This suggests that it may be appropriate to feed patients undergoing therapeutic hypothermia following cardiac arrest.
